# Choroidal Thickness in Different Patterns of Diabetic Macular Edema

**DOI:** 10.3390/jcm11206169

**Published:** 2022-10-19

**Authors:** Rida Amjad, Cheong-Ah Lee, Hafiz Muhammad Umer Farooqi, Hina Khan, Dong-Guk Paeng

**Affiliations:** 1 Department of Retina, Amanat Eye Hospital, Rawalpindi 46000, Pakistan; 2 Ocean & Biomedical Ultrasound Laboratory, Department of Ocean System Engineering, Jeju National University, Jeju 63243, Korea

**Keywords:** choroidal thickness, diabetic macular edema, EDI-SD-OCT, image processing

## Abstract

This observational study investigated the changes in choroidal thickness (ChT) in different patterns of diabetic macular edema (DME) based on image processing using enhanced-depth imaging spectral-domain optical coherence tomography (EDI-SD-OCT). Participants with ocular conditions affecting the fundus view, including retinal diseases, were excluded. After observing the patient’s medical record, multicolor fundus photos, thickness maps, and subtypes of DME were diagnosed according to the criteria reported by the Early Treatment Diabetic Retinopathy Study (ETDRS). Edema was classified as focal or diffuse and was subdivided into cystic macular edema (CME), CME with subretinal fluid (CME+), and spongy macular edema (SME). Image processing was performed on the B-scan images from SD-OCT to segment the choroid layer and obtain the choroid thickness. A total of 159 eyes of 81 patients (46 males and 35 females; 57.53 ± 9.78 years of age), and 57 eyes of 30 healthy individuals (age 57.34 ± 8.76 years) were enrolled in this study. Out of 159 eyes, 76 had focal macular edema (FME), 13 exhibited SME, and 51 presented CME. Among those with cystic macular edema, 19 eyes showed subretinal fluid (CME+). The average choroidal thickness in FME, diffuse SME, CME, and CME+ was 216.95 ± 52.94 µm, 243.00 ± 46.34 µm, 221.38 ± 60.78 µm, and 249.63 ± 53.90 µm, respectively. The average choroidal thickness in age-matched controls was 213.88 ± 45.60 µm. Choroidal thickness increases with the severity of edema; choroidal thickness was higher in diffuse macular edema than in FME. However, choroidal thickness increased in cystic macular edema with subretinal fluid (CME+).

## 1. Introduction

Diabetic macular edema (DME) is one of the most common etiologies of visual impairment across the globe [1]. Its pathogenesis involves increased retinal vascular permeability. The choroidal vasculature is responsible for the blood supply to the photoreceptor cells and retinal pigment epithelium; therefore, it plays a vital role in the metabolic exchange at the avascular fovea [2,3]. Choroidal dysfunction has been known to occur in various retinal and choroidal pathologies [4]. Moreover, choroidal thickness serves as a biomarker in the screening, diagnosis, prognosis, and intervention of various retinal and choroidal diseases, including central serous chorioretinopathy (CSCR), age-related macular degeneration (AMD), polypoidal choroidal vasculopathy (PCV), idiopathic macular hole (IMH), and diabetic macular edema (DME) [5,6,7,8].

Clinical and experimental analyses have proved similarities in histopathological changes in the choroidal and retinal vasculature of DME, such as ischemia, neovascularization, vascular hyperpermeability, extravascular hemorrhage, increased vascular tortuosity, microaneurysms, vascular dilatation, and stenosis [9,10]. Ischemia and vascular hyperpermeability due to protein leakage in the choroidal interstitium have been reported in patients with DME [11,12]. These changes may affect choroidal thickness as well [13]. The current literature provides conflicting evidence regarding changes in choroidal thickness in patients with DME using optical coherence tomography (OCT) technology. Young et al. reported an increase in choroidal thickness (ChT) with an increasing severity of diabetic maculopathy or retinopathy [14]. In contrast, Tarek et al. reported a decrease in choroidal thickness related to DME [15]. It is also an important consideration that DME may assume different patterns based on the location and extent of retinal thickening, namely diffuse and focal. Hence, it will be interesting to observe how the choroid behaves when these different patterns of macular edema are considered individually.

OCT is commonly used for the clinical diagnosis of diabetic macular edema [16]. Recent advances in OCT technology, such as enhanced-depth imaging and swept-source OCT, allow a deeper penetration of light into the various tissue layers of the eye, thus allowing increased visualization and improved structural analysis of the choroid and thickness estimates [17,18]. Ophthalmologists traditionally perform choroid thickness measurements using the caliper tool in commercial software. The sketch lines to calculate choroidal thickness from the Bruch’s membrane (BM) to the choroid outer boundary depend on individual experience and expertise [19,20]. From B-scan images, the outer boundary of the choroid termed choroid sclera junction is not often visible due to the heterogeneous characteristics of the choroidal tissue. Therefore, there could be an underestimation of the choroidal thickness and difference in values between observers. Various software have been designed to overcome this drawback by providing automated segmentation of OCT scans [21,22]. The existing literature reported using manual, semi-automatic, and automatic segmentation based on deep learning. However, to the best of our knowledge, automated analysis has not been used to measure and compare the choroidal thickness in different patterns of retinal edema, considering the Pakistani population [23].

The present study compared choroidal thickness among different DME patterns using manual segmentation (gold-standard method) and automated in-house-developed image-processing methods [24].

## 2. Materials and Methods

Prior written approval from the institutional review board (IRB) of Amanat Eye Hospital, Rawalpindi, Pakistan, was obtained before the data collection was started. The retrospective study enrolled 81 patients (46 males and 35 females; mean age 57.53 ± 9.78 years) with type II diabetes visiting Amanat Eye Hospital, Rawalpindi, Pakistan, from August 2021 to January 2022. The study included 159 eyes with DME. All the patients underwent complete ophthalmic examination, including trial refraction, best-corrected visual acuity measurements, slit-lamp biomicroscopy, and dilated fundoscopy as per the hospital routine and all data were recorded. Treatment-naive diabetic patients with DME who underwent multicolor fundus photography and OCT scans (Spectralis; Heidelberg Engineering, GmbH 2017, Heidelberg, Germany. SN: Catalogue numberCAM-05862-53610) were included in the study. Presence of high myopia or hyperopia (i.e., refractive error >±4.0 D and astigmatism of ±3.0 D), eyes with media opacities hampering fundal view and imaging (i.e., dense cataract), and retinal diseases other than diabetic retinopathy known to influence choroidal thickness (i.e., age-related macular degeneration, central serous retinopathy, retinal pigment epithelial detachment, branch retinal vein occlusion, central retinal vein occlusion, branch retinal artery occlusion, central retinal artery occlusion, hemiretinal vein occlusion, and uveitis) were excluded. Patients who had undergone anti-VEGF, periocular or intraocular steroid injection, or laser photocoagulation treatment were also excluded from the study. Participants with conditions including AMD, CSCR, and PCV in the fellow eye were excluded as these may affect choroidal thickness in the testing eye.

### 2.1. Diagnostic Criteria for DME

DME was defined as having central retinal thickness of more than 300 microns on OCT scan obtained from the available automated retinal thickness in the ETDRS grid sectors. The pattern of DME was confirmed from the thickness profile displayed in the Heidelberg Eye Explorer software. Macular volume (MV) and central retinal thickness (CRT) were also recorded in all eyes included in the study.

### 2.2. Classification of DME

**Figure 1:**
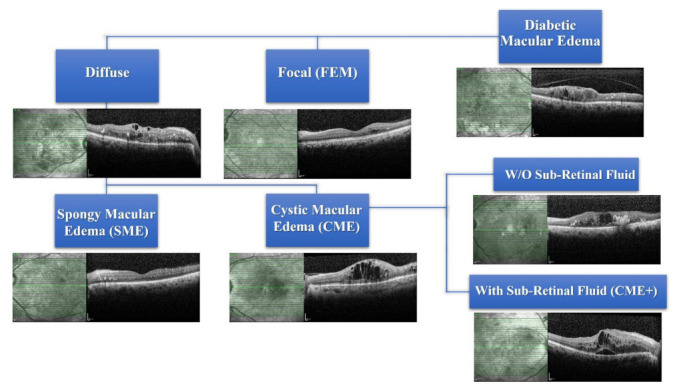
Classification of diabetic macular edema. The representative case showing the 4 types of diabetic macular edema (diffuse, focal, spongy macular edema, and cystic macular edema). In particular, cystic macular edema is segregated between without (W/O) subretinal fluid edema and with subretinal fluid edema (CME+).

Subtypes of DME were diagnosed according to the criteria reported by ETDRS by observing the patient’s medical record, multicolor fundus photos, and automated retinal thickness maps on SD-OCT scans [18,21]. Edema was classified into focal macular edema (FME) or DME. The diffuse macular edema was further divided into cystic macular edema (CME) and spongy macular edema (SME). CME is involved in eyes without (W/O) subretinal fluid (SRF) and with SRF. Amongst the eyes with CME, the presence of subretinal fluid (SRF) was labelled a “+” sign, i.e., CME+ (Figure 1). Macular volume (MV) and central retinal thickness (CRT) were also recorded in all eyes included in the study.

### 2.3. Choroidal Thickness Measurement by Manual Method

**Figure 2:**
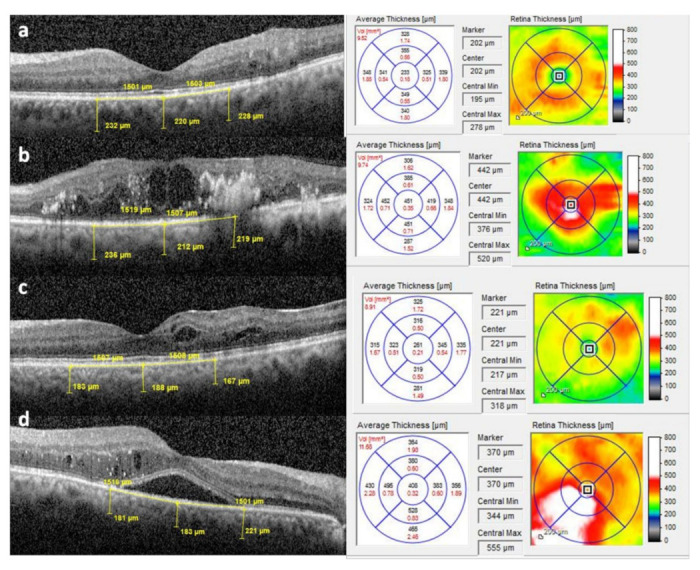
Measurement of choroidal thickness using calipers on a horizontal SD-OCT scan and thickness maps of (**a**) spongy macular edema (SME), (**b**) cystic macular edema (CME), (**c**) focal macular edema (FME), and (**d**) CME with subretinal fluid (SRF).

Heidelberg Eye Explorer software (Version 1.10.2.0) was used to measure choroidal thickness in the central and two parafoveal points on the horizontal B scan (as shown in Figure 2). Using the caliper tool in the OCT Heidelberg Eye Explorer software (Heidelberg Engineering), the vertical distance was measured at the fovea from the hyperreflective line of the Bruch’s membrane to the hyperreflective line of the chorio–scleral interface. Parafoveal choroidal thickness (PFChT) was measured at a manually calculated distance of 1500 µm from nasal and temporal to the foveal center using the same method stated above. Central retinal thickness (CRT) and macular volumes (MV) were automatically measured by in-house-developed software and recorded measurements were available. Control data were also obtained from the normal eyes of age-matched patients.

### 2.4. Choroidal Thickness Measurement Using Automated Image Processing

**Figure 3:**
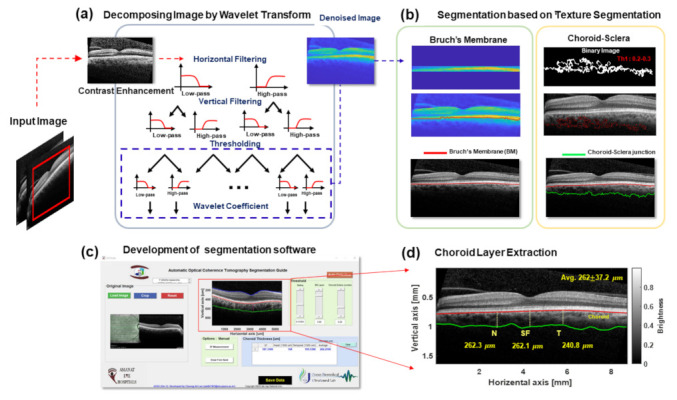
Schematic flow chart of the image-processing protocol from the B-scan image of an OCT scan. (**a**) Decomposing process based on wavelet transformation reduces image noise and enhances boundary layer visibility. (**b**) Segmentation process of BM and choroid layers. In particular, the binary image of the choroid layer texture is adopted to extract the choroid layer. (**c**) The algorithm of (**a**,**b**) are implemented to the guide user interface (GUI) using MATLAB software. The GUI is available to detect the retina, BM, and choroid layer and preserve the thickness of the choroid in three different locations (subfoveal, nasal, and temporal) and the average within the region of interest (ROI). (**d**) The automatically extracted results for the choroid layer.

The method opted for image processing is illustrated in Figure 3. The goals of image processing were to minimize personal error and save time in calculating the choroidal thickness. The image contrast of the OCT B-scan can be enhanced to increase the visibility of the boundary layers in the preprocessing step. To reduce the speckle noise of the image, original OCT images were reconstructed as denoised images using two-dimensional wavelet-transform-based filtering, as shown in Figure 3a,c, illustrating the BM boundary segmentation procedure [25,26]. The boundary of the BM layer was easily segmented due to the high intensity of brightness and homogeneous medium characteristics compared to the periphery layers in the OCT B-mode scan. Based on the BM segmentation, the inner layer of the choroid was extracted. After the BM boundary position, pixels corresponding to a specific threshold were extracted. The choroid layer texture was removed to acquire the outer layer of the choroid. The choroid layer is larger than other membranes, and several blood vessels are usually located within it. Hence, it was easy to extract the texture of the choroid layer. As shown in Figure 3c, the extracted textures were recorded as binary images and adopted by a designed spatial filter to detect choroid–sclera junctions. Finally, when both the BM and choroid layers were calculated to obtain the segmented information, the smooth filter was applied to eliminate any abrupt transitions of the boundary layers. The snapshot in Figure 3c highlights the developed GUI based on MATLAB software’s image-processing algorithm [27,28,29,30]. Ultimately, the retina, BM, and choroid layer segmentation was carried out. After segmenting each layer, the thickness was automatically calculated at three different locations (subfoveal (SF), nasal (N), and temporal (T)) at 1500 μm intervals, and the average thickness of the region of interest (ROI) is presented in Figure 3d.

## 3. Statistical Analysis

**Figure 4:**
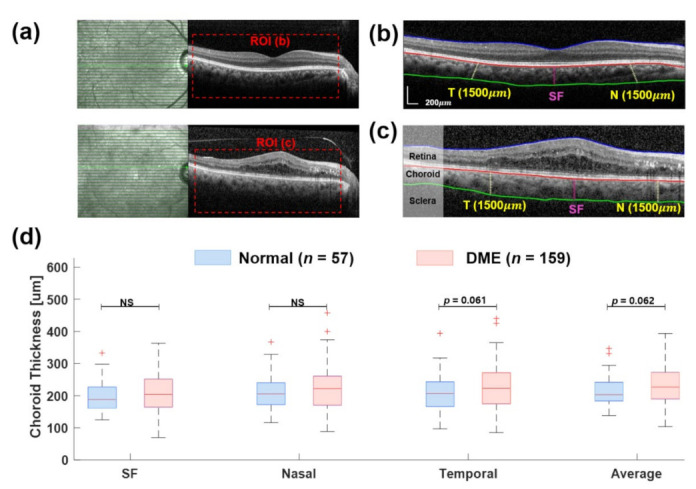
Comparing choroid thickness between normal and diabetic patients based on image automated retinal and choroidal thickness segmentation. (**a**) OCT image scans of two subjects with normal and diabetic macular edema (DME) eyes. Images processing perfumed at ROI (**b**,**c**). Example segmentation image of the normal eye (**b**) and DME (**c**) represent the retinal and choroid boundary layers. The solid lines correspond to the extracted boundary positions on the outer retinal surface (blue), BM (red), and choroid scleral junction (green). The vertical lines indicate the thickness of the choroid at 500 μm intervals from nasal (N) to temporal (T) to the subfoveal (SF). (**d**) Comparison of choroid thickness between control (*n* = 57) and DME eyes (*n* = 159) obtained at different three locations (SF, N, and T) and average choroid thickness of the whole area of ROI. Comparison data are shown in composite box plots. The *p*-value (*p*) is used in the independent *t*-test. (NS: not significant; statistically significant differences: *p* < 0.05).

Data were analyzed using the Statistical Package for the Social Sciences (SPSS) version 23.00 and MS Excel 2016 software. Data were presented as mean and standard deviation. For the quantitative variable, the normality assumption was evaluated by using the Shapiro–Wilk and Kolmogorov–Smirnov tests. ANOVA and independent-sample *t*-test were applied to compare choroidal thickness in diffuse and focal groups, and a post hoc test was performed for inter-group comparisons [31,32]. The *p*-value of ≤0.05 was considered significant. Two investigators independently extracted the thickness of choroid layers of the whole scan using implemented GUI, as shown in Figure 4.

## 4. Results

**Figure 5:**
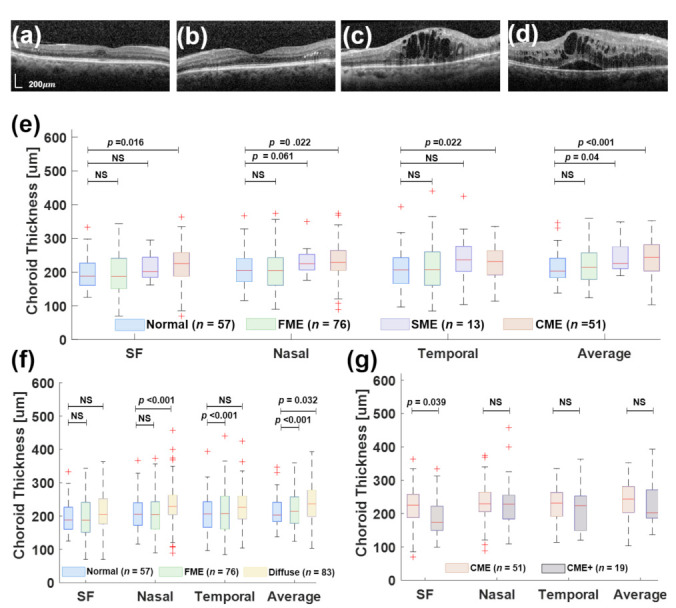
Measurement of choroidal thickness using the proposed in this work for segmentation of the retina and choroid layer. (**a**) Focal macular edema (FME). (**b**) Spongy macular edema (SME). (**c**) Cystic macular edema (CME). (**d**) CME with subretinal fluid (CME+). (**e**) Comparison of choroid thickness between normal, FME, SNE, and CME at different locations (SF, N, and T) and average choroid thickness. (**f**) Comparison of choroid thickness between normal and FME and Diffuse included SME and CME groups. (**g**) Comparison of choroid thickness between CME and CME+.

The mean duration of diabetes for the included patients was 14.2 years and HbA1C levels ranged from 7.9% to 12.2%. A considerable number of DME-affected eyes were found among diabetic patients. Out of 159 eyes from 81 diabetic patients with DME, 76 (47.77%) eyes with FME and 64 (40.25%) eyes with DME were identified. Out of 64 eyes with DME, 13 eyes (20.31%) had SME and 51 eyes (79.68%) showed CME (Figure 4). Among the 51 eyes with diffuse CME, 19 (37.25%) eyes had subfoveal fluid (CME+), as shown in Figure 5. The mean choroidal thicknesses (as measured using the manual method) in the central, nasal parafoveal, and temporal parafoveal points of the different subgroups defined earlier are shown in Table 1 and Table 2. It was observed that choroidal thickness tends to increase with the severity of edema, i.e., the choroid is thicker in eyes with diffuse macular edema (spongy or cystic) than in eyes with focal macular edema. Moreover, no significant difference was observed in the choroidal thickness between cystic edemaNo with SRF and without SRF.

Additionally, choroid thickness in the same subgroups of patients was observed through automated image segmentation (Figure 4 and Figure 5). Figure 4a shows the B-scan samples of OCT, where the red box indicates the ROI. There, two examples illustrate the segmentation process using wavelet-based image processing with texture extraction. The results of the choroid thickness measurement were obtained in three locations at 500 μm intervals, from the nasal (N) to the temporal (T) to the subfoveal (SF), with an average thickness of the whole ROI. In Figure 4d, the choroid thickness at the SF location of both normal (197.17 ± 49.5 μm) and DME (206.46 ± 62.16 μm) groups was thicker than in other locations. The average choroid thickness in the DME group was greater than the normal group (229.07 ± 54.5 μm vs. 213.88 ± 45.6 μm; *p* = 0.062, Table 3 and Table 4).

## 5. Discussion

Different patterns of edema represent varying degrees of severity regarding the leakage and pathology in diabetic patients. Studies show that patients with DME show a greater severity in leakage on fluorescein angiography (FA) as compared to eyes with SME. Similarly, eyes with FME show less leakage on FA than eyes with diffuse DME. Our study shows that the choroidal thickness not only increases in the presence of edema as compared to the controls, but that the magnitude of this increase in choroidal thickness also increases with the severity of the retinal pathology. The choroid was thicker in the eyes of CME than focal or spongy macular edema.

DME represents severe intraretinal microvascular pathology [33]. Yeung et al. demonstrated that the presence of cystic edema on OCT is correlated with more severe leakage on FA than spongy edema [34]. The present study indicates that the change in choroidal thickness also mirrors the severity of intraretinal microvascular pathology with the focal retinal thickening associated with less average ChT compared to both patterns of diffuse edema (cystic and spongy).

Studies on choroidal thickness in DME in published literature have conflicting results. We have observed that this conflict may be influenced by the selection criteria. Studies which include both treated and untreated eyes tend to report a decrease in ChT in DME [35,36]. Okomato et al. demonstrate the influence of the intraretinal treatments on ChT, i.e., anti-VEGF treatments and retinal laser photocoagulation (both causing choroidal thinning) [37,38]. Our results compared more directly to the eyes that follow a similar inclusion and exclusion criteria.

Systemic pathological conditions, such as hypertension and renal failure, have been shown to influence choroidal thickness [39]. The choroid is more amenable to change in acute settings. Studies have demonstrated a significant change in choroidal thickness after a single session of hemodialysis in patients with renal failure [40,41]. Other studies have also shown a change in choroidal thickness with fluctuation in plasma glucose levels. These findings indicate that the choroid is a more dynamic tissue than the retina [42,43,44]. However, our results show that the severity of microangiopathy is comparable in the retina and underlying choroid [45].

The pathophysiology of subfoveal fluid in DME is distinct. Subfoveal fluid has been speculated to develop due to leakage of proteins after ischemic events in the retina [46]. It has been shown that the central retina is predisposed to the formation of subretinal fluid in its anatomy as it has a high cell count, a Henle’s fiber layer, and a central avascular zone, creating a watershed pattern between choroidal and retinal circulation that decreases resorption of extracellular fluid [47]. Our study showed no significant difference with or without subretinal fluid regarding choroidal thickness. This indicates that the retinal factors may not directly influence the choroid in cases of SRF.

Although the relationship between retinal edema and underlying choroidal thickening remains unclear, there appears to be a common underlying microangiopathy driven by VEGF. Increased expression of the VEGF protein has been demonstrated in eyes with DME, and anti-VEGF treatment has decreased the ChT in eyes with DME [38]. The correlation seen in our study between the increasing severity of retinal edema and the choroidal thickness appears to be linked to pathological mechanisms common to both vascular tissues [48].

Two methods of ChT measurement have been employed separately in this study. The manual gold-standard method and the automated segmentation analysis have demonstrated consistent results across all edema patterns. In this study, the retina and choroid–scleral junction were automatically segmented using the in-house image-processing method based on wavelet transform filtering, with texture extraction of the choroid layer. It was observed that the automated software took less time to segment the choroid than manual analysis and could potentially decrease the task time problems in a clinical setting [49].

The limitation of our study is the small sample size, which could not be generalized to a bigger population. Further segmentation of choroidal layers (inner and outer layers) may be attempted in a larger sample size, and the effects of different pathologies could be observed independently between these layers. Additional avenues of research would include an analysis of how other systemic diabetic treatment options may affect ChT in eyes with DME.

## 6. Conclusions

The choroidal thickness varies with the severity of edema, and the choroid is thicker in diffuse macular edema (SME or CME) than in focal macular edema (FME). Furthermore, no difference was seen in the choroidal thickness between CME with SRF and without SRF.

## Figures and Tables

**Table 1:** Comparison of choroidal thickness measurements (by manual method) between spongy macular edema, cystic macular edema, and focal macular edema.

Choroidal Thickness (µm)	Spongy Macular Edema (*n* = 13)	95% of CI	Cystic Macular Edema (*n* = 51)	95% of CI	Focal Macular Edema (*n* = 76)	95% of CI	*p*-Value
Central position	253.69 ± 44.32	226.91–280.47	244.55 ± 58.39	228.13–260.97	243.43 ± 37.37	225.83–243.03	0.268
Nasal position	267.00 ± 47.11	238.53–295.47	243.37 ± 40.33	232.0–254.75	232.03 ± 43.92	221.92–242.13	0.020
Temporal position	271.77 ± 47.69	242.95–300.59	264.41 ± 51.09	250.04–278.78	249.52 ± 40.54	240.19–258.85	0.095
Average choroidal thickness	264.15 ± 40.03	239.96–288.34	250.77 ± 45.72	237.91–263.64	238.65 ± 35.49	230.49–246.82	0.055

**Table 2:** Comparison of choroidal thickness measurements (by manual method) between cystic macular edema and cystic macular edema having subretinal fluid.

Choroidal Thickness (µm)	Cystic Macular Edema without Subretinal Fluid (*n* = 32)	95% of CI	Cystic Macular Edema with Subretinal Fluid (*n* = 19)	95% of CI	*p*-Value
Central position	244.55 ± 58.39	228.13–260.97	246.53 ± 43.96	225.33–267.33	0.894
Nasal position	243.37 ± 40.33	232.00–254.75	246.79 ± 56.15	219.95–274.03	0.780
Temporal position	264.41 ± 51.09	250.04–278.78	268.47 ± 40.07	249.16–287.79	0.756
Average thickness	250.77 ± 45.72	237.91–263.64	253.92 ± 40.61	234.35–273.50	0.793

**Table 3:** Comparison of choroidal thickness measurements (using the automated method) between normal group and diabetic macular edema (DME) groups at three locations.

Choroidal Thickness (µm)	Normal (*n* = 57)	DME (*n* = 159)	*p*-Value *
Central position	197.17 ± 49.45	206.46 ± 62.16	NS (*p* = 0.31)
Nasal position 1500 μm (N)	208.00 ± 49.38	221.84± 65.49	NS (*p* = 0.14)
Temporal position 1500 μm (T)	206.46 ± 58.50	222.32± 71.09	NS (*p* = 0.13)
Average thickness	213.88 ± 45.60	229.07 ± 54.53	*p* = 0.06

Choroidal thickness: mean ± SD; SD: standard deviation; DME: diabetic macular edema. * The *p*-value using the independent *t*-test; NS: not significant; significant differences at *p* < 0.05.

**Table 4:** Comparison of choroidal thickness measurements (using the automated method) between normal and focal macular edema (FME), spongy macular edema (SME), cystic macular edema (CME), and CME with subretinal edema (CME+).

Choroidal Thickness (µm)	Normal (*n* = 57)	FME (*n* = 76)	SME (*n* = 13)	CME (*n* = 51)	CME+ (*n* = 19)
Central position	197.17 ± 49.45	198.65 ± 64.53	215.12 ± 41.67	223.33 ± 62.36	188.32 ± 64.98
Nasal position 1500 μm (N)	208.00 ± 49.38	208.25 ± 62.31	236.27 ± 43.13	245.89 ± 66.22	235.94 ± 87.02
Temporal position 1500 μm (T)	206.46 ± 58.50	212.24 ± 69.43	241.27 ± 77.49	229.73 ± 71.08	213.79 ± 67.42
Average thickness	213.88 ± 45.60	216.95 ± 52.94	243.00 ± 46.34	249.63 ± 53.90	221.38 ± 60.78

## Data Availability

The data presented in the study are available upon reasonable request from the corresponding authors.
